# Glycogen Synthase Kinase 3*β* Promotes Postoperative Cognitive Dysfunction by Inducing the M1 Polarization and Migration of Microglia

**DOI:** 10.1155/2020/7860829

**Published:** 2020-11-28

**Authors:** Jingjin Li, Chonglong Shi, Zhengnian Ding, Wenjie Jin

**Affiliations:** Department of Anesthesiology, The First Affiliated Hospital of Nanjing Medical University, Nanjing 210029, China

## Abstract

Postoperative cognitive dysfunction (POCD) is a common postoperative central nervous system complication, especially in the elderly. It has been consistently reported that the pathological process of this clinical syndrome is related to neuroinflammation and microglial proliferation. Glycogen synthase kinase 3*β* (GSK-3*β*) is a widely expressed kinase with distinct functions in different types of cells. The role of GSK-3*β* in regulating innate immune activation has been well documented, but as far as we know, its role in POCD has not been fully elucidated. Lithium chloride (LiCl) is a widely used inhibitor of GSK-3*β*, and it is also the main drug for the treatment of bipolar disorder. Prophylactic administration of lithium chloride (2 mM/kg) can inhibit the expression of proinflammatory mediators in the hippocampus, reduce the hippocampal expression of NF-*κ*B, and increase both the downregulation of M1 microglial-related genes (inducible nitric oxide synthase and CD86) and upregulation of M2 microglial-related genes (IL-10 and CD206), to alleviate the cognitive impairment caused by orthopedic surgery. In vitro, LiCl reversed LPS-induced production of proinflammatory mediators and M1 polarization of microglia. To sum up these results, GSK-3*β* is a key contributor to POCD and a potential target of neuroprotective strategies.

## 1. Introduction

POCD, a common postoperative complication, which is usually observed in elderly patients, refers to cognitive decline after surgery. POCD may persist for a long time and even develop into serious central nervous system diseases [1]. For the past few years, the incidence and even mortality of POCD have been increasing. Severe surgical trauma and advanced age are the two main risk factors for POCD [2]. Although the pathogenesis of POCD is not clear, there is growing evidence that neuroinflammation plays a crucial part in the disease [3]. Peripheral surgical trauma can bring about hippocampal-dependent learning and memory impairment, which are relevant to the increased level of IL-1*β* in the hippocampus [4]. The strategy of inhibiting hippocampal inflammation by blocking TNF-*α* can in turn prevent cognitive decline in aseptic surgical rat models [5].

In the central nervous system, inflammation is mainly controlled by microglia, a monocyte macrophage cell type that accounts for up to 20% of all brain cells. The aggregation and activation of microglia are related to the pathogenesis of Alzheimer's disease (AD), amyotrophic lateral sclerosis, and Parkinson's disease (PD). Activated microglia can release a variety of inflammatory mediators, including chemokines, cytokines, prostaglandins, excitatory amino acids, reactive oxygen intermediates, and nitric oxide [6]. Eventually, these inflammatory signals can enhance oxidative stress, activate the pathway of cell death, and promote neurodegeneration [7]. According to the activation state, microglia can be divided into classical activation (M1) and alternating activation (M2). Although this classification may be oversimplified, microglia are polarized into an activated state, an intermediate state between neurotoxicity and protection. M1 microglia can express proinflammatory factors including tumor necrosis factor-*α* (TNF-*α*), interleukin-1*β* (IL-1*β*), and nitric oxide (NO), as well as cell surface markers such as CD86, while M2 microglia express different factors, such as IL-4, arignase-1, YM1, CD206, and IL-10 [8].

GSK-3 is a highly conserved serine/threonine protein kinase, which is known to have *α* and *β* subtypes. Two subtypes of GSK-3 have extensive homology in the kinase domain, but may have distinct functions because of their unique N-terminal and C-terminal. Most studies on GSK-3 have focused on the *β* isoforms using chemical inhibitors, which do not distinguish between the *α* and *β* isoforms [9]. GSK-3*β* is constitutively active in resting cells and can be inhibited by Ser9 phosphorylation after stimulation. GSK-3*β* regulates different cellular activities in diverse cell types, including differentiation, proliferation, metabolism, apoptosis, and immune activation [10]. Inhibition of GSK-3*β* suppresses the proinflammatory gene program of macrophages, but promotes the anti-inflammatory gene program [11]. Toll-like receptor activation triggers GSK-3*β* to inhibit phosphorylation through phosphatidylinositol 3-kinase- (PI3K-) protein kinase B/AKT pathway, giving rise to an increase in cyclic AMP response element-binding protein (CREB) activity but a decrease in NF-*κ*B activity, which is a self-limiting mechanism for macrophages to avoid inappropriate inflammatory overactivation [12]. However, the relationship between microglial polarization and GSK-3*β* is still unclear. The role of GSK-3*β* in microglial polarization, neuroinflammation, and POCD is worthy of further investigation.

## 2. Materials and Methods

### 2.1. Animals

Male SD rats aged 16 months were purchased from Nanjing Medical University and used in this study (*n* = 24). All rats were housed in groups of four per cage with water and food available ad libitum. The ambient temperature of the housing and testing rooms was 22 ± 1°C. The rats were housed in a light-dark cycle of 12 h. The study was approved by the Nanjing Medical University Animal Care and Use Committee, and the experiments were performed according to the Guide for the Care and Use of Laboratory Animals of the National Institutes of Health of the United States.

### 2.2. Drug Treatment and Surgical Procedure

Aged rats were randomly divided into four groups: (a) intraperitoneal injection of normal saline (control group), (b) intraperitoneal injection of LiCl (Li group), (c) surgery following an intraperitoneal injection of normal saline (Op group), and (d) surgery following an intraperitoneal injection of LiCl (Li+Op group). Specifically, rats in the Li and Li+Op groups received an intraperitoneal injection of 2 mM/kg LiCl daily for 7 days [13], while the other groups received an equivalent volume of normal saline. On the 8^th^ day, all aged rats received sevoflurane anesthesia (1.7% inspired concentration at 0.4 FiO_2_) and analgesia with buprenorphine (0.08 mg/kg s.c.) with or without surgery. To verify the role of GSK-3*β* in the present models, we also examine other GSK-3*β* inhibitor, such as SB-216763. In the same way as LiCl, rats received an intraperitoneal injection of 2 mg/kg SB216763 daily for 7 days.

**Figure 1:**
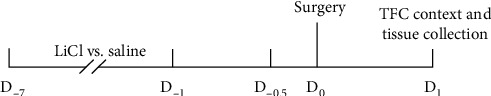
Study design. The aged rats were divided into four groups and received the lithium or saline treatment for 7 consecutive days. Then, the rats in the appropriate group received the tibia fracture surgery. The TFC training was performed 30 min before surgery. The brain tissues and serum samples were collected 24 h after surgery. Behavioral tests were also performed at this time point.

As previously described, surgical animals underwent an open tibial fracture of the right hind paw with an intramedullary fixation under anesthesia [14]. In short, under completely aseptic conditions, the right hind limb of each surgical animal was meticulously shaved and disinfected with povidone iodine. A middle incision was performed on the right hind knee followed by the insertion of a 20-G pin in the intramedullary canal, the periosteum was then stripped, and osteotomy was performed. After producing the fracture, the wound was irrigated before the skin was sutured with 8/0 Prolene sutures. Temperature maintained at 37°C with a heating pad throughout the surgical process. The study design is briefly illustrated in Figure 1.

### 2.3. Cell Cultures

Primary rat microglia were prepared as previously described, with sight modifications [15]. In short, the whole brain tissues of postnatal days one to two from SD rats were triturated. The dissociated cells were passed through a 100 *μ*m pore mesh, pelleted by centrifugation at 1500 rpm for 5 min, and resuspended in culture medium. Cells were seeded on poly-D-lysine-precoated cell culture flasks in high-glucose Dulbecco's Modified Eagle's Medium (DMEM, Gibco, USA) containing 10% fetal calf serum, 100 U/ml penicillin, and 100 mg/ml streptomycin (Gibco, USA). Cultures were maintained at 37°C in a humidified atmosphere of 5% CO_2_/95% air. After the glial cells formed a confluent monolayer (10-14 days), the microglial cells were separated from the astrocytes by shaking for 5 h at 110 rpm. The microglial cells were seeded into 6-well culture plates at a density of 10^5^ cells/cm^2^. After 24 h of culture, the cells were starved overnight and then subjected to treatments. The primary microglia were pretreated with LiCl (1 mM) for 30 min and then incubated with LPS (10 ng/ml) for 24 h. The purity of the microglia was confirmed to be >98% using immunofluorescence staining for OX-42 (Abcam, Hong Kong, China) and was calculated as follows: number of OX − 42 − positive cells/number of DAPI − positive cells.

### 2.4. Trace Fear Conditioning (TFC)

TFC was used to evaluate hippocampus-dependent memory in rodents as previously described [16]. The clear acrylic TFC chamber (Xeye Fcs, Beijing MacroAmbition S&T Development Co., Ltd., Beijing, China), with dimensions of 30 cm long, 30 cm wide, and 30 cm high, included a floor constructed of stainless steel bars that was connected to a shock delivery system. Training and assessment were performed in a room illuminated with overhead fluorescent bulbs with a ventilation fan providing background noise (65 dB). During training, an initial exploratory phase (100 s) was followed by two trials separated by a 100 s intertrial interval. Trials consisted of a 20 s auditory cue (80 dB and 5 kHz, conditional stimulus), followed by a 2 s foot shock (0.8 mA, unconditional stimulus). Rats anticipate the shock by “freezing,” which is defined as the absence of all movements expects for respiration; this defensive posture reflects learned fear. When placed in the same context on a subsequent occasion, the learned fear is recalled and the amount of learning and recall is measured by the amount of freezing. Memory of the learned fear was assessed 1 day after the surgery by returning the rat to the same chamber in which it was trained, in the absence of tone and shock. Freezing behavior was automatically scored for 300 s by the video tracking software (Xeye Fcs).

### 2.5. Immunohistochemistry

Rats were deeply anesthetized and perfused transcardially with 200 ml of normal saline followed by 200 ml of 4% paraformaldehyde. The brains were immersed in 30% sucrose for 48 h. Sections were then incubated with the Iba1 polyclonal antibody (1 : 200) at 4°C overnight and then incubated with secondary antibody for 2 h. Microglial cells were visualized by adding DAB to the sections. Activated microglia were identified as Iba1-positive cells. Iba1-positive cells were counted in a blinded fashion by an experimenter who was unaware of the sample identity. For quantification, the studied tissue sections were selected with a 150 *μ*m interval according to anatomical landmarks corresponding to Bregma from Bregma −2.8 to −3.8 mm of the rat brain (Paxinos and Watson, 1996). For each animal, 9 photographs from the CA1 area of three hippocampus sections were captured using Leika 2500 (Leica Microsystems, Wetzlar, Germany) at 200x magnification. The number of Iba1-positive cells per photograph (0.74 mm^2^ frame) was obtained by using the NIH ImageJ software (Bethesda, MD, USA), averaged and converted to cells/mm^2^. Iba1-positive cell counting was performed in a blinded fashion by an experimenter that was unaware of the sample identity.

### 2.6. ELISA

Concentrations of TNF-*α* and IL-1*β* in brain tissue extracts were measured with ELISA kits from R&D Systems (Minneapolis, MN, USA).

### 2.7. Western Blotting

Hippocampi were homogenized in a lysis buffer containing 10 mM EGTA, 5 mM EDTA, a proteinase inhibitor cocktail, 20 mM PNPP, 1 mM Na_3_VO_4_, 30 mM *β*-glycerophosphate, and 0.05 mM NaF. The homogenates were centrifuged at 12000g for 20 min (4°C), and the supernatants were then harvested as cytosolic fractions for the immunoblot analysis. The pellet was resuspended in a lysis buffer that also contained 10% Nonidet P 40, 10% Brij-35, and 10% sodium deoxycholate for the detection of nuclear proteins or with a lysis buffer that also contained 10% SDS for the detection of membrane proteins. After an oscillation on ice for 60 min, the solutions were centrifuged at 7900g for 10 min (4°C), and the supernatants were nuclear proteins or membrane proteins. The protein concentration of each supernatant was determined using a BCA protein assay kit. Proteins (60 *μ*g) were denatured with SDS sample buffer and separated using 10% SDS-polyacrylamide gel electrophoresis (PAGE). The proteins were transferred to a polyvinylidene fluoride (PVDF) microporous membrane (Millipore, Bedford, MA), which was then blocked with 5% skim milk for 1 h at room temperature. The membrane was incubated with a primary antibody overnight at 4°C. The following primary antibodies were used: rabbit polyclonal antibodies against p-GSK-3*β*, GSK-3*β*, NF-*κ*B p65, GAPDH, and histones (Cell Signaling Technology, USA). After incubation with the anti-rabbit secondary antibody for 1 h, the protein bands on the membranes were detected with ECL kits (Thermo Fisher Scientific, USA). The relative density of the protein bands was scanned by densitometry using the Image Lab software (Bio-Rad, Richmond, CA, USA) and quantified using the NIH ImageJ software (Bethesda, MD, USA).

### 2.8. Real-Time PCR

Total RNA was extracted from hippocampi and primary microglial cells using Trizol reagent (Invitrogen, USA), and reverse transcription was performed with the Transcription First Strand cDNA Synthesis Kit (Roche, Switzerland) according to the manufacturer's protocol. The cDNA templates were prepared from 1 *μ*g of total RNA using a PrimeScript RT Master Mix kit (TaKaRa Bio, Japan) according to standard protocols. Quantitative PCR was performed on a StepOnePlus Real-Time PCR System (ABI, USA) using the synthetic primers and SYBR Green (TaKaRa Bio, Japan). Primers were as follows: CD86 forward, AGCCCACGTCGTAGCAAACCAC; CD86 reverse, AGGTACAACCCATCGGCTGGCA; iNOS forward, GGGAGCCAGAGCAGTACAAG; iNOS reverse, TGCAGATTCTGGAGGGATTT; IL-10 forward, GGCAGAGAACCATGGCCCAGAA; IL-10 reverse, AATCGATGACAGCGCCTCAGCC; CD206 forward, TCAGCTATTGGACGCGAGGCA; CD206 reverse, TCCGGGTTTGCAAGTTGCCGT. Samples were subjected to 40 cycles of amplification at 95°C for 5 s and 60°C for 30 s, after incubation at 95°C for 30 s. Relative expression was calculated using the 2 − ^(Ct experimental sample–Ct internal control sample (GAPDH))^ method.

### 2.9. Transwell Migration Assay

Transwell migration assays were performed using 8 *μ*m pore diameter inserts (Corning, Lowell, MA). Briefly, 2 × 10^4^ microglia cells were plated in the upper chamber with 200 *μ*L serum-free medium. This upper chamber was then placed within the bottom wells containing 600 *μ*L conditioned medium. LiCl (1 mM) was added to the medium of the upper chamber for 30 min. Then, LPS (10 ng/ml) was added to the bottom wells for 24 h. Following incubation at 37°C for 24 h, nonmigrating cells on the upper surface of the membrane were carefully removed with a cotton swab. Cells on the lower surface of the membrane were first fixed in 4% paraformaldehyde for 30 min, followed by staining with 0.2% crystal violet for 1 h. For quantification, six randomly chosen fields on the lower membrane surface were imaged using computer-assisted microscopy.

### 2.10. Flow Cytometry Analysis

Flow cytometry was employed to determine CD86 (BD Biosciences, USA) expression in rat microglia. The dissociated cells from hippocampal tissues were incubated with FITC-conjugated mouse anti-CD86 antibody and PE-conjugated mouse anti-OX-42 antibody or isotype control (1 : 200) for 1 h at 37°C. FACS Calibur flow cytometer (BD Biosciences, USA) was used to analyze the cells.

### 2.11. Statistical Analysis

All experiments were performed in triplicate. Statistical analyses were performed using the GraphPad Prism 5 software (version 5.01, GraphPad Software, San Diego, CA). The results are presented as means ± s.e.m. Data were analyzed using one-way ANOVA followed by a post hoc test when appropriate. A *P* value of <0.05 was considered statistically significant.

## 3. Results

### 3.1. Inhibition of GSK-3*β* Alleviates Tibial Surgery-Induced Memory Impairments

**Figure 2:**
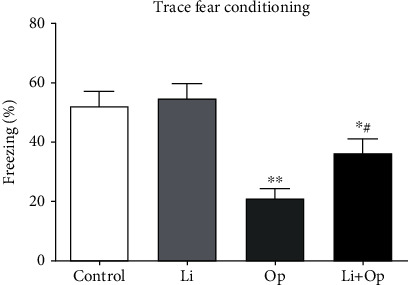
Inhibition of GSK-3*β* alleviates tibial surgery-induced memory impairments. Contextual fear response, as measured by freezing behavior, was determined in the rats. The data are presented as means ± s.e.m. (*n* = 6 rats). ^∗^*P* < 0.05 and ^∗∗^*P* < 0.01 compared with the control group. ^#^*P* < 0.05 compared with the OP group.

One day after the tibial surgery, we performed contextual fear assessments to observe the cognitive function of the senile rats. As shown in Figure 2, the rats exposed to tibial surgery exhibited a significant reduction in cognitive function compared to animals exposed to saline. The prophylactic lithium treatment significantly improved freezing behavior, indicating that LiCl attenuated the memory dysfunction caused by surgery (Figure 2). Based on these results, tibial surgery impairs the cognitive function of aged rats, and GSK-3*β* inhibition limits the adverse cognitive outcomes caused by tibial surgery.

### 3.2. Inhibition of GSK-3*β* Suppresses Tibial Surgery-Induced Microglial Activation and Inflammatory Response

**Figure 3:**
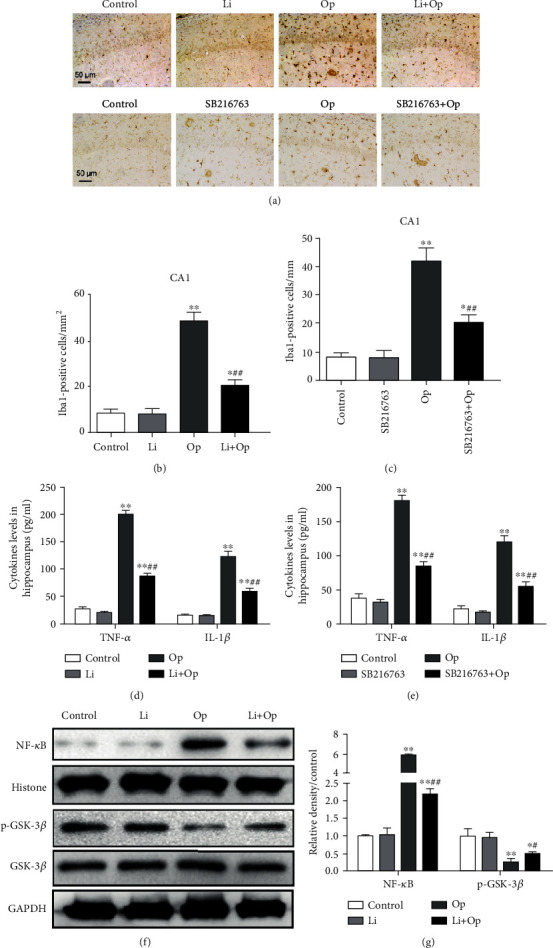
Inhibition of GSK-3*β* suppresses tibial surgery-induced microglial activation and inflammatory response. (a) Representative images of immunohistochemical staining of microglia in the CA1 area of the hippocampus. (b, c) Quantification of Iba1-positive cells in the CA1 area of the hippocampus. (d, e) Levels of the TNF-*α* and IL-1*β* proteins in the hippocampus were determined using ELISAs. (f) Levels of NF-*κ*B and p-GSK-3*β* in the rat hippocampus were detected using Western blotting with specific antibodies. (g) The levels of proteins were quantified by ImageJ, and the levels of p-GSK-3*β* and NF-*κ*B were normalized to GSK-3*β* or histone levels (p-GSK-3*β*/GSK-3*β* ratio and NF-*κ*B/histone ratio). Each value was then presented relative to the ratio of control, which was set to 1. The data are presented as means ± s.e.m. (*n* = 3). ^∗^*P* < 0.05 and ^∗∗^*P* < 0.01 compared with the control group. ^#^*P* < 0.05 and ^##^*P* < 0.01 compared with the OP group.

Tibial surgery induced an increase in the number of Iba1-positive cells in the CA1 area of the hippocampus. The microglia exhibited an enlarged cytoplasm and cell bodies, irregular shapes, and intensified Iba1 staining, consistent with the morphological characteristics of activated microglia (Figures 3(a)–3(c)). This effect was significantly inhibited by the GSK-3*β* inhibitor, prophylactic lithium, or SB216763, suggesting that GSK-3*β* inhibition suppresses the microglial activation induced by tibial surgery.

Levels of the TNF-*α* and IL-1*β* in the hippocampus were detected using ELISAs to determine whether the tibial surgery induced neuroinflammation in aged rats. As shown in Figures 3(d) and 3(e), one day after tibial surgery, the levels of TNF-*α* and IL-1*β* in hippocampus were significantly higher than those in the control group. The LiCl or SB216763 pretreatment partially abolished the increase in tibial surgery-induced TNF-*α* and IL-1*β* production. Thus, GSK-3*β* inhibition reduces neuroinflammation induced by the tibial surgery.

The Western blot analysis shows significantly lower levels of the inhibitory phosphorylation of GSK-3*β* in the operative rats than the control group, and this change was reserved by LiCl (Figures 3(f) and 3(g)). NF-*κ*B activation might initiate an inflammatory cascade, leading to an increased release of proinflammatory cytokines. Surgery increased the expression of NF-*κ*B, while the LiCl or SB216763 pretreatment inhibited this effect, suggesting that GSK-3*β* inhibition restrains NF-*κ*B pathway.

### 3.3. Effects of GSK-3*β* Inhibition on the Expression of M1 and M2 Markers in Microglia

**Figure 4:**
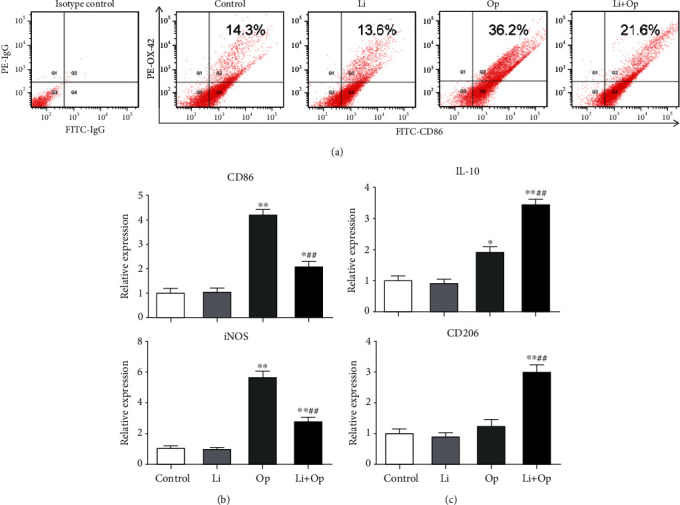
Effects of GSK-3*β* inhibition on the expression of M1 and M2 markers in microglia. (a) Flow cytometry analysis was used to determine the CD86 expression in hippocampal microglia. (b, c) Expression of M1 (CD86 and iNOS) and M2 (IL-10 and CD206) markers in the hippocampus was examined using quantitative RT-PCR. The data are presented as means ± s.e.m. (*n* = 3). ^∗^*P* < 0.05 and ^∗∗^*P* < 0.01 compared with the control group. ^##^*P* < 0.01 compared with the OP group.

The expression of M1 markers (CD86, iNOS, TNF-*α*, and IL-1*β*) was significantly increased in the surgery group, while the LiCl pretreatment partially abolished this change (Figures 3(d), 4(a), and 4(b)). Remarkably, the LiCl pretreatment also increased the expression of M2 markers (IL-10 and CD206) (Figure 4(c)), suggesting that GSK-3*β* inhibition induces the polarization of microglia from the M1 to M2 phenotype.

### 3.4. LPS-Induced Microglia Activation and Migration Are Suppressed by GSK-3*β* Inhibition *in vitro*

**Figure 5:**
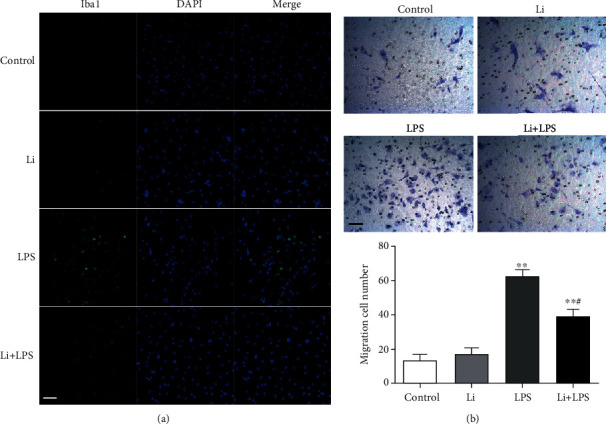
LPS-induced microglia activation and migration are suppressed by GSK-3*β* inhibition *in vitro*. The primary microglia were pretreated with LiCl (1 mM) for 30 min and then incubated with LPS (10 ng/ml) for 24 h. (a) Activation of primary microglia was examined using immunofluorescence. The microglia cells were stained with Iba1 antibody. Expression of Iba1 expression (*green*) in activated microglia was visualized by confocal microscopy. The blue staining represents DAPI. Scale bar = 50 *μ*m. (b) Crystal violet staining of primary microglia that migrated into the lower surface of the polycarbonate membrane inserts (8 *μ*m pore size) at 24 h after seeding. Scale bar = 50 *μ*m. Graph demonstrates the average number of migrating cells per visual field in six random fields. The data are presented as means ± s.e.m. (*n* = 3). ^∗^*P* < 0.05 and ^∗∗^*P* < 0.01 compared with the control group. ^#^*P* < 0.05 compared with the LPS group.

To confirm the effects of GSK-3*β* inhibition on microglia activation and migration in vitro, primary microglia were stimulated with LPS. The primary microglia were pretreated with LiCl (1 mM) for 30 min and then incubated with LPS (10 ng/ml) for 24 h. As shown in Figure 5(a), after incubation with LPS for 24 h, the Iba1 expression in microglia was remarkably upregulated compared with the expression observed in the control group. However, pretreatment with LiCl remarkably inhibited the effect of LPS. These results suggest that LPS could induce the activation of microglia, which can be suppressed by LiCl.

As shown in Figure 5(b), the number of migrating cells was increased after incubation with LPS in comparison to the number of control group. However, pretreatment with LiCl remarkably reduced the number of migration cells when compared with control group (Figure 5(b)). These results suggest that GSK-3*β* may lead to recruitment of microglial cells.

### 3.5. LPS-Induced Expression of M1 Markers Is Suppressed by GSK-3*β* Inhibition *in vitro*

**Figure 6:**
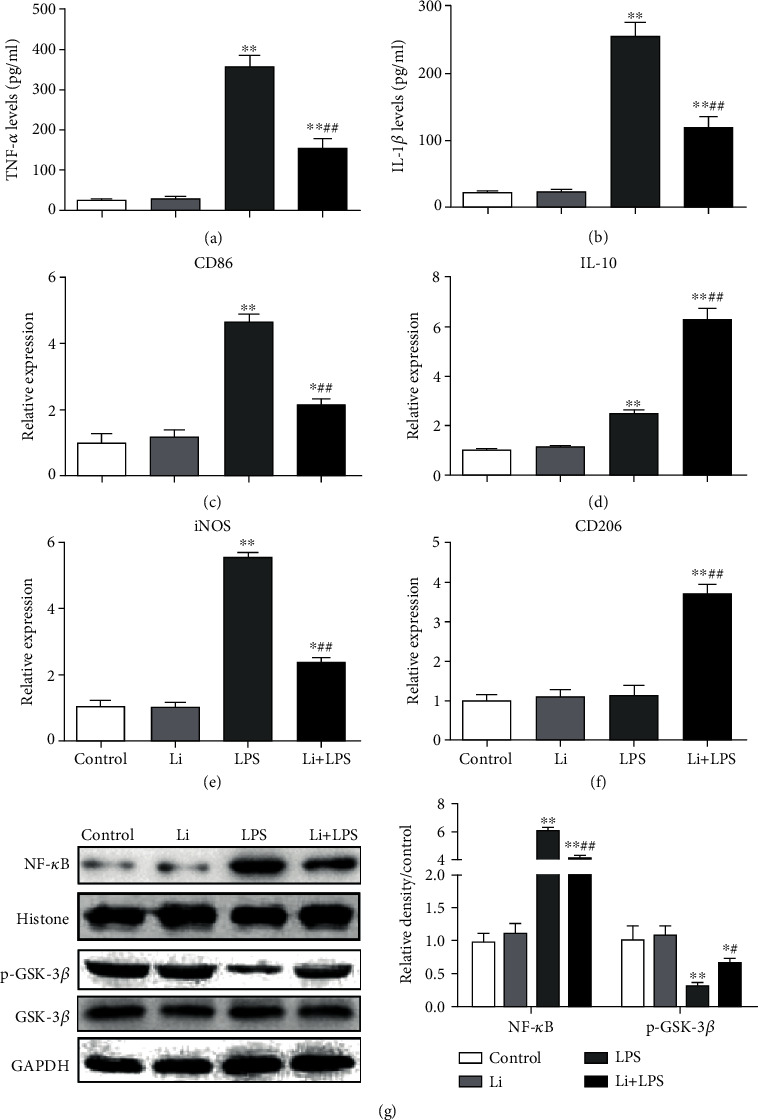
LPS-induced expression of M1 markers is suppressed by GSK-3*β* inhibition *in vitro.* (a–f) Expression of M1 (TNF-*α*, IL-1*β*, CD86, and iNOS) and M2 (IL-10 and CD206) markers in primary microglia was examined using ELISAs or quantitative RT-PCR. (g) Levels of NF-*κ*B and p-GSK-3*β* in the rat hippocampus were detected using Western blotting with specific antibodies. The levels of proteins were quantified by ImageJ, and the levels of p-GSK-3*β* and NF-*κ*B were normalized to GSK-3*β* or histone levels (p-GSK-3*β*/GSK-3*β* ratio and NF-*κ*B/histone ratio). Each value was then presented relative to the ratio of control, which was set to 1. The data are presented as means ± s.e.m. (*n* = 3). ^∗^*P* < 0.05 and ^∗∗^*P* < 0.01 compared with the control group. ^#^*P* < 0.05 and ^##^*P* < 0.01 compared with the LPS group.

According to the expression profile of M1 and M2 markers *in vivo*, we speculated that LiCl, a GSK-3*β* inhibitor, affected M1 polarization and M2 polarization. We examined whether the LPS-induced expression of M1 markers was inhibited by the LiCl treatment using primary cultures of microglia to address this hypothesis. The expression of M1 markers (TNF-*α*, IL-1*β*, CD86, and iNOS) was significantly upregulated by LPS and attenuated by the cotreatment with LiCl. Coincident with the changes in M2 markers *in vivo*, cotreatment with LiCl also increased the expression of M2 markers (IL-10 and CD206) (Figures 6(a)–6(f)).

LPS stimulates the NF-*κ*B pathway. Activated NF-*κ*B upregulates the expression of inflammatory genes. We examined the LPS-NF-*κ*B axis to investigate the mechanism of GSK-3*β* inhibition. NF-*κ*B expression was upregulated by LPS and inhibited by cotreatment with the GSK-3*β* inhibitor *in vitro* (Figure 6(g)).

## 4. Discussion

Peripheral surgeries in POCD animal models have been shown to bring about cognitive impairment and neuroinflammation, which are similar to the symptoms observed in humans [17]. The purpose of this study was to investigate the cognitive effects and neuroinflammatory profiles after tibial fracture surgery in aged rats. Cognitive effects were evaluated using the TFC test, which is a technique widely used in behavioral neuroscience to study cognition [15]. In our study, aged rats that underwent surgery showed cognitive impairments. Meanwhile, compared with rats that only underwent surgery, pretreating with GSK-3*β* inhibitor LiCl prior to surgery improved the freezing behavior and the number of learning trials. The above results indicate that the spatial learning and memory of rats were maintained after surgery.

Persistent neuroinflammation plays a significant role in the occurrence and development of POCD, which has been identified as a pathological mechanism of POCD [18]. Increased levels of proinflammatory molecules in systemic circulation and central nervous system have been observed in elderly patients after hip surgery [19]. Similarly, peripheral surgical wounding induces the systemic release of proinflammatory cytokines and neuroinflammation, which lead to cognitive impairment in aged rats [17]. Under the stimulation of surgery, a large number of proinflammatory cytokines are released to induce neuroinflammation, which has been postulated to mediate the pathology of POCD. In the present study, the levels of IL-1*β* and TNF-*α* were observed significantly increased in the hippocampus of rats that underwent surgery. When rats were administered with LiCl prior to surgery, the increase of IL-1*β* and TNF-*α* was inhibited. TNF-*α* is located upstream of IL-1*β* and can stimulate the production of IL-1*β* in the hippocampus. TNF-*α* may enter the brain through relatively permeable areas of the blood-brain barrier [5]. Peripheral blockade of TNF-*α* can inhibit the production of IL-1*β*. Cibelli et al. confirmed that surgery caused postoperative memory dysfunction was related to increased plasma cytokines, as well as reactive microgliosis and IL-1*β* expression in the hippocampus [5, 20].

Microglia are thought to be the tissue macrophages of the brain. In the central nervous system, microglia are pivotal in immune surveillance, which are primed and easily respond to the peripheral stimulation induced by injury [21]. Accumulating studies have reported strong evidence that microglia play a dual role, both beneficial and harmful. On one hand, the activation of microglial is thought to benefit the damaged brain by removing cellular debris and restoring tissue integrity (M1) [22]. On the other hand, activated microglia can release proinflammatory mediators, such as TNF-*α*, nitric oxide, and IL-1*β*, leading to neuronal dysfunction and cell death (M2) [23]. In the pathophysiological process, microglia expressed the “beneficial” M2 phenotype in the early stage and were gradually replaced by the “harmful” M1 phenotype without any therapeutic intervention. In the present study, the LiCl pretreatment not only inhibited the expression of the “harmful” M1 phenotype stimulated by surgery or LPS, but also induced the expression of the “beneficial” M2 phenotype. It has been found that GSK-3*β* plays a key role in microglial polarization, but LiCl alone had no effect on microglial polarization. LiCl did not directly affect the “resting” microglia, but induced the transition from M1 to M2 in the process of neuroinflammation. The role of GSK-3*β* in regulating TLR-dependent signaling in response to a variety of agonists, including LPS, has been previously studied in peripheral monocytes/macrophages, astrocytes, and dendritic cells. According to the results of these *in vitro* and *in vivo* studies, constitutively active GSK-3*β* induces the expression of proinflammatory cytokines and chemokines, whereas it inhibits the production of the anti-inflammatory cytokine IL-10. Consistent with these findings, the inhibitor of GSK-3*β* can attenuate the expression of LPS-dependent proinflammatory genes and enhances the expression of IL-10 [24]. In the present study, the inhibitor of GSK-3*β* suppressed the activation of microglia induced by LPS, which was associated with a transition from the M1 to M2 phenotype.

Taken together, the results of this study indicated that peripheral tibial fracture surgery induced the change of cognitive in aged rats, which was accompanied by M1 polarization of microglia. Meanwhile, GSK-3*β* inhibition could improve cognitive decline by inducing M2 polarization of microglia, indicating that this protein is a potentially important target for the treatment of postoperative cognitive dysfunction in elderly patients.

## Data Availability

The data used to support the findings of this study are available from the corresponding authors upon request.
